# Activated Protein C Does Not Alleviate the Course of Systemic Inflammation in the APCAP Trial

**DOI:** 10.1155/2012/712739

**Published:** 2012-05-08

**Authors:** Lea Kyhälä, Panu Mentula, Leena Kylänpää, Eeva Moilanen, Pauli Puolakkainen, Ville Pettilä, Heikki Repo

**Affiliations:** ^1^Department of Surgery, Helsinki University Central Hospital, 00029 Helsinki, Finland; ^2^The Immunopharmacology Research Group, University of Tampere School of Medicine and Tampere University Hospital, 33521 Tampere, Finland; ^3^Intensive Care Units, Department of Anesthesia and Intensive Care Medicine, Helsinki University Central Hospital, 00029 Helsinki, Finland; ^4^Department of Medicine, University of Helsinki, 00029 Helsinki, Finland

## Abstract

The study aimed to determine the effect of the activated protein C on the course of systemic inflammation in the APCAP (activated protein C in acute pancreatitis) trial where we randomized 32 patients with severe acute pancreatitis to receive either recombinant activated protein C (drotrecogin alfa activated) (*n* = 16) or placebo (*n* = 16) for 96 hours. In the present study, we present the time course of the patients' plasma or serum levels of soluble markers (IL-8, IL-6, IL-10, IL-1ra, sE-selectin, PCT) and monocyte and neutrophil cell surface (CD11b, CD14, CD62L, HLA-DR) markers of systemic inflammatory response during the first 14 days after the randomization. The results of the intervention and placebo groups were comparable showing that recombinant APC treatment did not alter the course of systemic inflammation in severe acute pancreatitis. Our finding is in accordance with the clinical findings in the APCAP trial indicating that the intervention did not affect evolution of multiple organ dysfunctions.

## 1. Introduction

Acute pancreatitis (AP), a common cause of abdominal pain, is usually a mild, self-limited disease. However 25% of the patients suffer from severe AP (SAP) [1], and 20% of SAP patients die, [2], mostly due to the development of multiple organ dysfunction [3]. Systemic inflammation, typical of AP, is considered to contribute to the development of organ dysfunction. It is characterized by (i) an increase in circulating levels of proinflammatory cytokines [4, 5], anti-inflammatory cytokines [6–9], and soluble E-selectin (sE-selectin) [10–12], a marker of activation of the vascular endothelium, (ii) occurrence of activated phagocytes in the circulation [13, 14], and (iii) a decrease in HLA-DR expression on blood monocytes [9, 13, 15, 16], denoting the development of immune suppression.

Activated protein C (APC) is a plasma serine protease with effects on coagulation, apoptosis, and inflammation [17]. APC acts as an endogenous anticoagulant that promotes fibrinolysis and inhibits thrombosis. Protein C, an inactive precursor, is converted to activate protein C by thrombin-thrombomodulin complex on endothelium [18]. This process is accelerated in the presence of endothelial PC receptor (EPCR) [19]. APC inactivates the procoagulation factor Va and VIIIa shutting down the coagulation pathway. APC also inactivates plasminogen activator inhibitor, which results in increased fibrinolysis [18].

APC also has cytoprotective effects such as anti-inflammatory, antiapoptotic, and endothelial barrier protection effects [20].

Proinflammatory cytokines upregulate thrombin formation and downregulate the host's antithrombotic mechanisms, in particular the protein C (PC) pathway reviewed in [21]. Deficiency of PC and decreased generation of activated PC (APC), the major endogenous anticoagulant in man, associate with the development of organ dysfunction in AP [22]. In patients with sepsis human recombinant APC shortened the duration of respiratory dysfunction and accelerated the reversal of shock [23]. We studied in a randomized trial patients with severe AP and found no differences in the evolution of multiorgan dysfunction between APC and placebo groups [24]. However, the effects of APC on inflammatory markers in patients with SAP have not been studied in a randomized controlled trial previously.

Accordingly, we aimed to determine the effects of the APC intervention on plasma levels of proinflammatory (IL-8), pro-/anti-inflammatory (IL-6) and anti-inflammatory (IL-10, IL-1ra) [25] cytokines and sE-selectin, on activation markers of blood monocytes (CD14, CD11b, CD62L) and neutrophils (CD11b, CD62L), on levels of monocyte cell-surface expression of HLA-DR, a marker of immune suppression, and on serum levels of procalcitonin (PCT), a marker of systemic inflammation used in clinical decision making.

## 2. Subjects and Methods

### 2.1. Patients and Healthy Subjects

We previously conducted a randomized study of APC in SAP patients [24]. In brief, this prospective randomized double-blind study included analysis of 32 patients with SAP in the tertiary care unit at the Helsinki University Central Hospital between June 2003 and August 2007. The inclusion criteria were (1) admitted to hospital <96 h from the onset of pain, (2) a 3-fold increase in serum amylase (IU/L) over normal upper range or/and verification of SAP in computer tomography, (3) at least one organ dysfunction (OD) defined as the Sequential Organ Failure Assessment (SOFA) of at least 3 of 4, and (4) <48 hours from the first OD. Patients were randomized to receive either APC (drotrecogin alfa activated) (*N* = 16) or 0.9% physiologic saline as placebo (*N* = 16). APC was administrated for 96 hours with a dose of 24 *μ*g/kg/hour.

We obtained reference blood samples for the analyses of cell surface markers by flow cytometry from 65 healthy volunteers (137 samples) from the hospital and laboratory staff without medication and with no signs of infection. To monitor the level of fluorescence intensity, a blood sample from a healthy volunteer was studied according to the study protocol once a week. There were 58 women and 7 men in the reference group. In case of repeated sampling, mean of the data was used.

The study protocol was approved by the ethics committees of the Helsinki University Central Hospital. Informed consent was obtained from all patients or their next of kin. The study protocol was registered in ClinicalTrials.gov (NCT01017107).

### 2.2. Blood Samples

When a patient fulfilled the inclusion criteria we collected peripheral blood samples for determination of cell markers of inflammation by venipuncture for the first time. Then the patients were randomized. After that follow-up samples were collected in the morning of the third, fifth, seventh, and 14th day. Blood samples for flow cytometry and for plasma measurements were anticoagulated with pyrogen-free acid-citrate dextrose (ACD). Blood samples were immediately cooled in an ice-cold water bath and kept at 0°C until stained for flow cytometry. The plasma was separated by centrifugation at +4°C and stored at −70°C until concentrations of cytokines and sE-selectin were determined. Blood samples for determination of serum PCT were collected concurrently.

### 2.3. Analysis of Soluble Markers

The concentrations of IL-6, IL-8, IL-10, IL-1Ra, and E-selectin in plasma samples were determined by enzyme immunoassay (EIA) by using commercial reagents (IL-6 and IL-10: PeliPair ELISA, Sanquin, Amsterdam, the Netherlands; IL-8: Opt EIA, BD Biosciences, Erembodegem, Belgium; IL-1Ra: Duo Set ELISA, R&D Systems Europe Ltd, Abindgon, UK; E-Selectin: ELISA, HyCult Biotechnology, Uden, The Netherlands). The detection limits and intra-assay and interassay coefficients of variation (CV%) were as follows: IL-6: 0.3 pg/mL, 3.6% and 5.4%; IL-8: 1.6 pg/mL, 3.5%, 3.4%; IL-10: 0.3 pg/mL, 3.7%, 5.9%; IL-1Ra: 10 pg/mL, 4.2%, 5.2%; E-selectin 20.5 pg/mL, 3.5%, 6.9%.

Procalcitonin (PCT) was measured using ADVIA Centaur XP immunoassay system with ADVIA Centaur BRAHMS PCT assay. Assay is a sandwich chemiluminescent immunoassay using monoclonal antibody to fluorescein covalently linked to paramagnetic particles and two antibodies to procalcitonin labelled with fluorescein. According to the manufacturer, the within-run precision of the method is 4.3%, 1.5%, and 1.5% for PCT at 0.2, 0.97, and 65.9 *μ*g/L, respectively. The between-run precision is 8.5%, 2.1%, and 7.2% for the respective concentrations. The limit of detection for the assay is 0.04 *μ*g/L and dilution point 75 *μ*g/L.

### 2.4. Analysis of Cell Surface Markers

**Monoclonal Antibodies and Flow Cytometry**

Monocyte expression of CD14, CD11b, CD62L and HLA-DR and neutrophil expression of CD62L and CD11b were determined using whole blood flow cytometry, as described previously [9, 13]. Monoclonal antibodies (mAbs) were as follows: phycoerythrin (PE) and fluorescein isothiocyanate (FITC) conjugates of anti-CD14 mAb (IgG2b, clone MFP9), PE conjugates of anti-HLA-DR mAb (IgG2a, clone L243), anti CD11b mAb (IgG2a, clone D12) and control mouse IgG2a, mAb, and FITC conjugate of anti-CD62L mAb (IgG2a, clone SK11). All reagents were purchased from Becton Dickinson (San Jose, CA, USA). Staining of aliquots of the whole blood sample at 0°C for flow cytometry was carried out as described previously [9, 13]. Data acquisition and analyses were done by a FACSCalibur flow cytometer and Cell Quest software (BD Sciences, San Jose, CA). Neutrophils were identified by the light scattering properties and monocytes by the clonal marker CD14. Monocyte HLA-DR expression was determined as the proportion of HLA-DR positive monocytes, as described earlier [13]. Fluorescence intensity is presented as relative fluorescence units (RFUs).

### 2.5. Statistical Analysis

The primary end point of the randomized study was the change in SOFA score. The sample size for the study was determined according to the primary end point: there would be three-point difference in change of SOFA score between the groups (with *P* < 0.05 and a power of 80%) [24]. Values are given as medians and ranges. Comparisons of marker levels between the two groups (the APC group and the placebo group) were performed by the Mann-Whitney *U*-test. In case of repeated sampling from the healthy volunteers mean data was used to get one value for each person and after that median was used. The Wilcoxon signed-rank test was used in comparisons of repeated measurements. A difference with a *P*-value of less than 0.05 was considered to be statistically significant. Statistical analysis was performed using SPSS19.0 statistical software (Chicago, Illinois).

## 3. Results

### 3.1. Patients

Characteristics of the 32 SAP patients are given in Table 1. All except one of the patients were admitted to the ICU. The time before patients were admitted to the ICU was 1,0 days (0–3 days) in APC group and 2,0 (1-2 days) in the placebo group (*P* = 0.642). In the APC group there were two nonsurvivors: one after receiving 13 hours of APC infusion and the other one having a laparotomy after 41 hours of APC infusion.

### 3.2. Soluble Markers

Plasma concentrations of proinflammatory cytokine IL-8 of all patients decreased during the first five days after the admission to hospital (day 0: 264 pg/mL versus day 5: 110 pg/mL, *P* = 0.001). The APC treatment had no significant effect on the changes in IL-8 concentrations during the follow-up period (Table 2).

**Figure 1:**
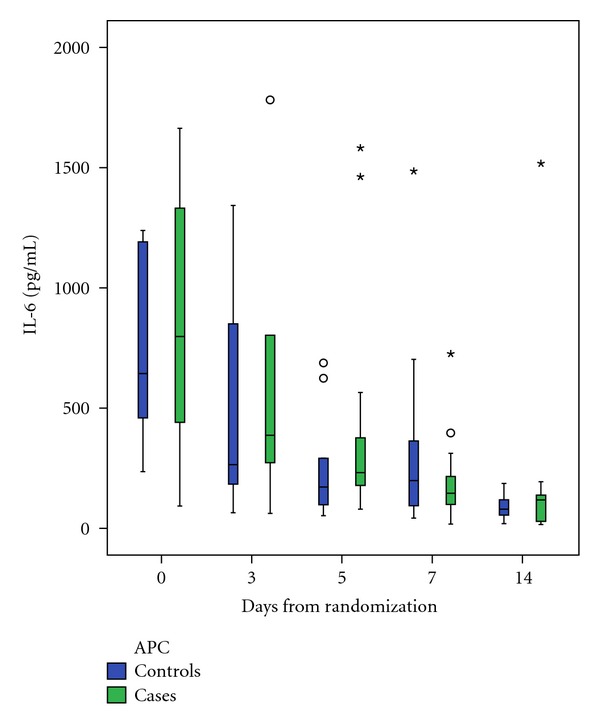
The changes in concentrations of IL-6 during the follow-up time. The APC did not have significant effect on the changes (*P* = 0.288). Box-Whisker plots show median, interquartile range (box) and highest and lowest values. Outliers (circles) and extreme values (asterisks) are shown separately.

Plasma concentrations of pro-/anti-inflammatory cytokine IL-6 (Figure 1), anti-inflammatory cytokines IL-10 and IL-1Ra of all patients decreased during the first five days of the follow-up time (IL-6 day 0: 670 pg/mL versus day 5: 215 pg/mL, *P* = 0.001; IL-10 day 0: 12.7 pg/mL versus day 5: 11.3 pg/mL, *P* = 0.001; IL-1Ra day 0: 2890 pg/mL versus day 5: 1250 pg/mL, *P* = 0.007). The APC treatment did not have any significant effect on the changes in IL-6, IL-10, and IL-1Ra concentrations during the first five or 14 days of follow-up time (Table 2).

Plasma concentrations of soluble E-selectin of all patients decreased along the course of the disease (day 0: 45.5 ng/mL versus day 5: 38.2 ng/mL, *P* = 0.031), but the APC treatment did not have any significant effect on the changes in concentrations of sE-selectin (Table 2).

There were no significant changes in serum concentrations of procalcitonin of all patients during the first five days (day 0: 0.97 ng/mL versus day 5: 0.66 ng/mL, *P* = 0.487), and administration of APC did not alter the changes in PCT concentrations (Table 2).

### 3.3. Cell Surface Markers

As a marker of immune suppression monocyte HLA-DR expression of all patients was not altered significantly during the first five days of follow-up period (day 0: 54% versus day 5: 58%, *P* = 0.316). Neither had the APC infusion any effect on the HLA-DR expression (Table 3).

The cell surface expressions of CD11b, CD14, and CD62L were measured as markers of activation of monocytes. The cell surface expression of CD11b, CD14, and CD62L on monocytes of all patients was downregulated during the first five days (MoCD11b day 0: 291 RFU versus day 5: 200 RFU, *P* = 0.001; MoCD14 day 0: 168 RFU versus day 5: 150 RFU, *P* = 0.028; MoCD62L day 0: 217 RFU versus day 5: 135 RFU, *P* = 0.001). The expression of CD11b, CD14, and CD62L did not differ significantly between the placebo and the APC-treatment group (Table 3).

**Figure 2:**
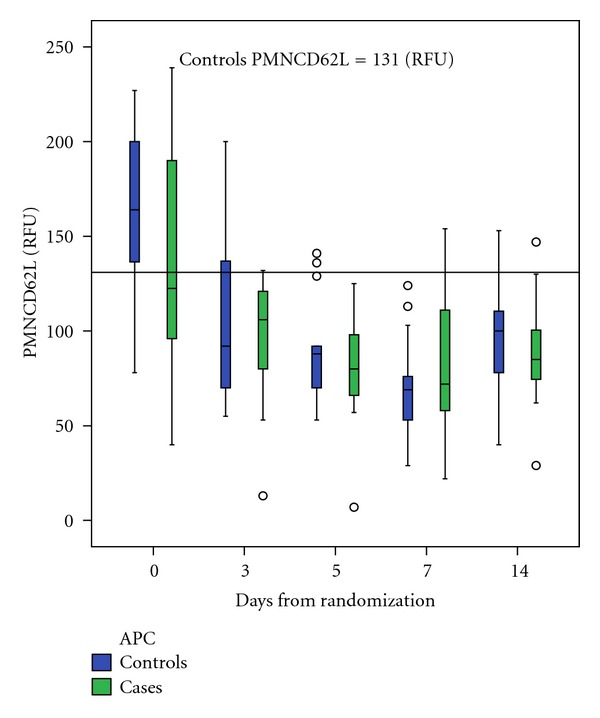
The changes in expression of CD62 on neutrophils during the follow-up time. The APC did not have significant effect on the changes (*P* = 0.251). Box-Whisker plots show median, interquartile range (box) and highest and lowest values. Outliers (circles) are shown separately.

The expressions of CD11b and CD62L were measured as markers of neutrophil activation. The expressions of CD11b and CD62L (Figure 2) of all patients were both downregulated during the first five days of follow-up period (PMNCD11b day 0: 325 RFU versus day 5: 259 RFU, *P* = 0.001; neutrCD62L day 0: 146 RFU versus day 5: 81 RFU, *P* = 0.001). The APC treatment did not have any effect on the changes in CD11b or CD62L expression on PMN cells between days 0 and 5 (Table 3).

## 4. Discussion

The results show that recombinant APC (drotrecogin alfa activated) treatment of patients with SAP did not alter the course of systemic inflammation, as determined using soluble and cellular markers of systemic inflammatory response. This is in accordance with the clinical findings of these patients, which indicated that SOFA score changes, organ-failure-free days, ICU or hospital stay time, ventilator-free days, renal replacement therapy-free days, vasopressor-free days, or days alive outside hospital (in 60 days) were comparable between APC and placebo groups [24]. The overall decreasing tendency of cytokines during the followup resembles earlier results, which show that both pro- and anti-inflammatory bursts are an early phenomenon in severe AP [15].

Several in vitro and animal studies show that APC has an anti-inflammatory activity. Administration of APC has been shown to downregulate the expression of inflammatory cytokines and chemokines. APC blocks cytokine production from Th2 lymphocytes [26]. APC has been shown to reduce production of endotoxemia-induced proinflammatory cytokines (IL-6, IL-8, IL-1beta, and TNF alfa) [27]. In vitro the production of IL-8 from monocytes is inhibited by APC (LPS-stimulated THP-1 cells) [28]. In vitro APC has been shown to inhibit chemotaxis and IL-6 release by human neutrophils [29]. APC inhibits TNF-alpha production by blocking nuclear factor (NF) kB transcription factor in monocytes [30]. APC has been shown to upregulate anti-inflammatory mediators, like IL-10 in blood monocytes in patients with severe sepsis [31]. APC can also block leukocyte trafficking by decreasing the expression of adhesion molecules (ICAM-1, E-selectin, and VCAM-1) on the endothelium [32–35].

Several clinical trials have been established to evaluate the APC treatment in sepsis patients. In the PROWESS trial they found that recombinant human activated protein C (drotrecogin alfa) reduced mortality in patients with severe sepsis [36]. In this randomized multicentre trial they found decreases in D-dimer levels and IL-6 levels in patients' plasma that has been taken as evidence of anti-inflammatory action of APC [23]. HLA-DR expression, as marker of immunosuppression, has been shown to correlate with PC and APC levels in SAP [22]. Preliminary, still unpublished results from the PROWESS-SHOCK trial indicated that APC did not have a beneficial effect on 28-day survival in patients with septic shock.

No previous randomized trial has scrutinized the effect of APC in SAP patients, nor is there such evidence of APC's effect on inflammation in SAP. The present study of phlogistic markers supports the view that APC treatment does not affect either the course of systemic inflammation in agreement with no detected effect on organ dysfunction [24].

*Limitations of the study.* The sample size for the study (16 + 16) was determined according to the primary end point and not for comparing systemic inflammatory response between the groups. In addition the number of follow-up samples/patients was decreased at day five (12 + 14) and at day 14 (11 + 10); therefore it is not possible to exclude a type II error.

## 5. Conclusion

The recombinant APC (drotrecogin alfa activated) treatment of patients with SAP did not alter the course of systemic inflammation, as determined using soluble and cellular markers of systemic inflammation.

## Figures and Tables

**Table 1:** Characteristics of patients.

	Activated protein C	Placebo
No. of patients	16	16
Male/female	16/0	15/1
Etiology of SAP -Alcohol/biliary	16/0	15/1
Age (years)	44 (34–36)	47 (19–59)
SOFA score on admission	8.0 (3–13)	8.5 (3–15)
ICU stay (days)	10.0 (2–43)	11.0 (0–31)

*Values are median (range).

**Table 2:** The changes in concentrations of soluble inflammation markers between days 0–5 and 0–14. Median (range).

Marker	APC baseline *n* = 16	Placebo baseline *n* = 16	*P*-value	APC change 0–5 d *n* = 14	Placebo change 0–5 d *n* = 13	*P*-value	APC change 0–14 d *n* = 11	Placebo change 0–14 d *n* = 11	*P*-value
IL-8, pg/mL	286 (56.4–1760)	245 (41.5–1750)	0.867	−72.3 (−1630–60.8)	−39.5 (−275–470)	0.159	−84.9 (−266–263)	−132 (−1280–−50.4)	0.270
IL-6, pg/mL	798 (93–7190)	643 (235–41800)	1.000	−377 (−1350–−1090)	−517 (−41600–−182)	0.159	−724 (−1550–979)^5^	−884 (−41700–−388)	0.288
IL-1ra, pg/mL	4480 (143–107000)	2870 (656–12400)	0.590	−2210 (−20500–2160)	−1480 (−1150–−6380)	0.627	−1500 (−7850–3950)	−750 (−3890–2240)	0.243
IL-10, pg/mL	13.7 (2.78–367)	10.8 (4.17–145)	0.724	−5.51 (−28.9–6.04)	−6.72 (−137–28.1)	0.438	−6.70 (−33.2–72.1)	−6.20 (−139–77.0)	0.898
sE-selectin, ng/mL	41.4 (25.3–195)	86.1 (19.0–192)	0.445	0.06 (−139–20.0)	−40.5 (−135–16.9)	0.099	0.100 (−143–14.4)	−45.6 (−144–8.70)	0.270
PCT, ng/mL	1.03 (0.12–8.33)^1^	0.75 (0.18–3.96)^2^	0.880	0.18 (−7.53–3.47)^3^	−0.21 (−1.91–1.62)^4^	0.590	0.130 (−4.66–3.96)	−0.270 (−3.30–2.09)^6^	0.918

^1^ *n* = 14, ^2^ *n* = 15, ^3^ *n* = 11, ^4^ *n* = 10, ^5^ *n* = 12, ^6^ *n* = 10.

d: day; IL: interleukin; PCT: procalcitonin.

*P*-values are calculated for differences between the two groups in changes from baseline.

**Table 3:** The changes in concentrations of cell markers between days 0–5 and 0–14. Median (range).

Marker median, range of healthy reference subjects, *n*	APC baseline *n* = 16	Placebo baseline *n* = 15	*P*-value	APC change 0–5 d *n* = 14	Placebo change 0–5 d *n* = 12	*P*-value	APC change 0–14 d *n* = 11	Placebo change 0–14 d *n* = 10	*P*-value
MoHLA-DR, % 95.0 (42–99), *n* = 64	54.0 (13–73)	44.0 (26–75)	0.599	10.0 (−21–30)	1.00 (−50–22)	0.173	13.0 (−18–37)	31.0 (−29–65)	0.152
MoCD11b, RFU 126 (72–297), *n* = 63	284 (118–770)	337 (136–673)	0.953	−91.5 (−290–123)	−76.5 (−348–114)	0.877	−35.0 (−301–445)	−15.0 (−497–150)	0.349
MoCD14, RFU 206 (74–379), *n* = 64	161 (87–306)	186 (96.0–294)	0.626	−28.5 (−115–68)	−21.5 (−142–58)	0.797	−5.00 (−75–19)	−59.5 (−119–60)	0.173
PMNCD11b, RFU 130 (75–466), *n* = 63	324 (172–791)	325 (161–519)	0.861	−50.5 (−426–236)	−66.0 (−219–164)	0.918	−37.0 (−215–242)	−74.0 (−346–89)	0.173
MoCD62L, RFU 118 (63–279), *n* = 64	217 (133–313)	216 (87–343)	0.892	−65.5 (−208–15)	−68.0 (−226–18)	0.979	−95.5 (−212–11)	−109 (−212–8)	0.481
PMNCD62L, RFU 131 (85–193) *n* = 64	123 (40–239)	164 (78–227)	0.264	−46.0 (−212–43)	−67.5 (−123–25)	0.898	−76.0 (−165–69)	−80.0 (−165–65)	0.251

Mo: monocyte; PMN: neutrophil; RFU: relative fluorescence units.

## Abbreviation

APCAP: Activated protein C in acute pancreatitis
AP: Acute pancreatitis
SAP: Severe acute pancreatitis
PC: Protein C
APC: Activated protein C
PCT: Procalcitonin
OD: Organ dysfunction
SOFA: Sequential organ failure assessment
EIA: Enzyme immuno assays
RFU: Relative fluorescence units
ICU: Intensive care unit
PROWESS: Recombinant Human Activated Protein C Worldwide Evaluation in Severe Sepsis.
